# Single and simultaneous effects of acrylamide and ethanol on bone microstructure of mice after one remodeling cycle

**DOI:** 10.1186/s40360-019-0317-7

**Published:** 2019-07-01

**Authors:** Anna Sarocka, Veronika Kovacova, Radoslav Omelka, Birgit Grosskopf, Edyta Kapusta, Zofia Goc, Grzegorz Formicki, Monika Martiniakova

**Affiliations:** 1 Department of Zoology and Anthropology, Constantine the Philosopher University in Nitra, 949 74 Nitra, Slovakia; 2 Department of Botany and Genetics, Constantine the Philosopher University in Nitra, 949 74 Nitra, Slovakia; 3 Institute of Zoology and Anthropology, Georg-August University, 37 073 Göttingen, Germany; 4 Department of Animal Physiology and Toxicology, Pedagogical University of Cracow, 30 084 Cracow, Poland

**Keywords:** Acrylamide, Ethanol, Cortical bone, Trabecular bone, Liver, Mice

## Abstract

**Background:**

This study aimed to examine femoral bone microstructure of mice after single and simultaneous administration to acrylamide and ethanol since both substances are often consumed separately and/or together by humans. Interactive effects of these toxins were analysed after one remodeling cycle.

**Methods:**

Twenty clinically healthy adult mice were randomly divided into four groups following 2 weeks administration of toxins: A group - mice were fed with acrylamide (40 mg/kg bw); E group - mice were ethanol-fed (15% ethanol); AE group - mice were simultaneously fed with both toxins, and a C group – control (without acrylamide and/or ethanol supplementation). Generally, 2D and 3D imaging methods were used to determine cortical and trabecular bone tissues microstructure. Biochemical analyses of plasma parameters were also realized using commercially available ELISA tests and spectrophotometrically.

**Results:**

Single and simultaneous exposure to acrylamide and ethanol affected only cortical bone microstructure. No significant changes in trabecular bone morphometry were detected among all groups. In mice from the A group, increased endocortical remodeling associated with a higher level of serum calcium and vasoconstriction of primary osteon’s vascular canals (POVC) were identified. On the contrary, increased cortical porosity consistent with a decreased relative bone volume, bone mineral density (BMD) and lower levels of alkaline phosphatase (ALP), glutathione (GSH), calcium in plasma and also with vasodilation of POVC were observed in the E group. In the AE group, the highest density of secondary osteons associated with a lower BMD and decreased levels of ALP, GSH were documented. The parameters of POVC and Haversian canals approximated to the C group. In addition, single and simultaneous exposure to both toxins caused liver disease consistent with a higher values of alanine aminotransferase (ALT), aspartate aminotransferase (AST) in plasma of all experimental groups.

**Conclusions:**

Single administration to acrylamide and ethanol had negative effects on cortical bone structure of mice after one remodeling cycle. However, we identified possible antagonistic impact of these toxins on the structure of the cortical bone.

## Background

Bone is a highly specialized supporting framework of the body, characterized by its rigidity, hardness, and power of regeneration and repair [1]. It is composed of support cells (osteoblasts and osteocytes), remodeling cells (osteoclasts) and non-mineral matrix of collagen and noncollagenous proteins (osteoid), with inorganic mineral salts deposited within the matrix [2]. During life, bones undergo processes of longitudinal and radial growth, modeling and remodeling [3]. Bone remodeling involves the removal of mineralized bone by osteoclasts followed by the formation of bone matrix through the osteoblasts that subsequently become mineralized. It serves to adjust bone architecture to meet changing mechanical needs and it helps to repair micro damages in bone matrix. Bone remodeling also plays an important role in maintaining plasma calcium homeostasis [4]. In general, the bone is able to accumulate various toxins from environment [5]. Therefore, physical activity and good nutrition are important factors which affect bone remodeling.

Acrylamide (AA), α,β-unsaturated reactive molecule, is an odorless crystalline solid [6]. Beviews its utilization in industry, AA is a neurotoxin that can be formed in some foods during high-temperature cooking processes, such as frying, roasting and baking [7]. In addition to its neurotoxic effect, AA is classified as a probable human carcinogen [8] with other toxic effects like genotoxicity [9] and reproductive toxicity [10]. It can also be absorbed across the skin [9].

Ethanol is not an essential nutrient but is largely consumed around the world also [11]. Its consumption affects the brain, liver, muscles and bones [12]. It is known that chronic heavy ethanol administration negatively modifies bone remodeling, including decreased number and activity of osteoblasts [13], modulation of Wnt signaling pathway which is responsible for regulation of bone mass [11] and increased oxidative stress [14].

In our previous studies, single acrylamide and/or ethanol administration in the diet negatively affected murine bone microstructure after 48 h and/or 4 remodeling cycles, respectively [15, 16]. Here we report the interactive effects of both toxins on bone microstructure of mice after one remodeling cycle (2 weeks).

## Methods

### Animals

In our experiment, 20 clinically healthy 12-weeks-old Swiss mice were used. We used the males because they are generally less susceptible to skeletal damage than females. Mice were fed a standard diet (Agropol, Motycz, Poland) and water ad libitum and grown in 12/12 light photoperiods. Animals were randomly segregated into four groups following 2 weeks administration of toxins: A group - mice were fed with acrylamide (40 mg/kg bw); E group - mice were ethanol-fed (15% ethanol); AE group - mice were simultaneously fed with both toxins, and the C group – control (without acrylamide and/or ethanol supplementation). The dose of AA was chosen on the basis of experiments conducted by other authors [17–19]. The dose of ethanol corresponds to a consumption of six 5 cl of 40% ethanol or 2.5 l of 12° beer for 75 kg male adults. Both toxins were dissolved in physiological saline and were administered orally to mice using a syringe in known doses. Animals from C group received only physiological saline solution. All the applied procedures were approved by the First Local Ethic Committee on Experiments on Animals in Cracow (resolution number 154/2014).

### Procedures

At the end of treatment period, mice were put into a state of deep anesthesia for sacrifice by Vetbutal (Biowet, Poland) administration in the amount of 35 mg/kg bw followed by rapid cervical dislocation and decapitation. Their femurs were next used for microstructural analyses. Each femur was macerated, degreased and embedded in epoxy resin Biodur (Gunter von Hagens, Heidelberg, Germany) according to the methodology of Martiniakova et al. [20]. Transverse thin sections (70–80 μm) were prepared with a sawing microtome (Leitz 1600, Leica, Wetzlar, Germany) and affixed to glass slides with Eukitt (Merck, Darmstadt, Germany) [21]. The qualitative 2D characteristics of the cortical bone were determined according to the internationally accepted classification systems of Enlow and Brown [22] and Ricqles et al. [23]. The quantitative 2D parameters of the cortical bone were assessed using software Motic Images Plus 2.0 ML (Motic China Group Co., Ltd.) in all views (*cranial, caudal, medialis, lateralis*) of thin sections. We measured area (μm^2^), perimeter (μm), mean diameter (μm) of the primary osteons’ vascular canals, Haversian canals and secondary osteons (all osteons were intact) in all views of thin sections in order to minimize statistical differences in the individual. Secondary osteons were distinguished from primary osteons (i.e., primary vascular canals) on the basis of the well-defined peripheral boundary (cement line) between secondary osteons and surrounding tissue. Generally, cement line delimits secondary osteons and also Haversian canals and does not outline primary osteons or their vascular canals [21, 24–26].

Quantitative 3D analyses of cortical and trabecular bone tissues were determined using microcomputed tomography (μCT 50, Scanco Medical). Cortical bone structure was analysed in a region of interest starting 5.2 mm from the end of the growth plate (distal end) and extending 1.5 mm at femoral midshaft. High resolution scans with a voxel size of 6.8 um were acquired (70 kV, 200 μA, 300 ms, 0.5 mm, aluminum filter). Following parameters were measured: relative bone volume (%), bone mineral density (BMD) (mg HA/ccm), bone surface (mm^2^) and cortical bone thickness (mm). Trabecular bone structure was analysed in a region of interest starting 1.2 mm from the end of the growth plate (distal end) and extending 1.5 mm. We measured relative bone volume (%), BMD (mg HA/ccm), trabecular number (1/mm), trabecular thickness (mm) and bone surface (mm^2^).

The activity of plasma bone ALP, ALT, AST, GSH and calcium (Ca) were measured using commercially available ELISA tests and spectrofotometrically.

### Statistics

Statistical analysis was performed using SPSS 17.0 software. The measured values were expressed as mean ± standard deviation. The differences in quantitative 2D and 3D parameters of cortical and trabecular bone tissues among mice from all groups were determined using Games-Howell’s and/or Tukey’s tests. The *P*-value less than 0.05 was considered to be statistically significant.

## Results

### Qualitative 2D analysis of cortical bone tissue

**Fig. 1:**
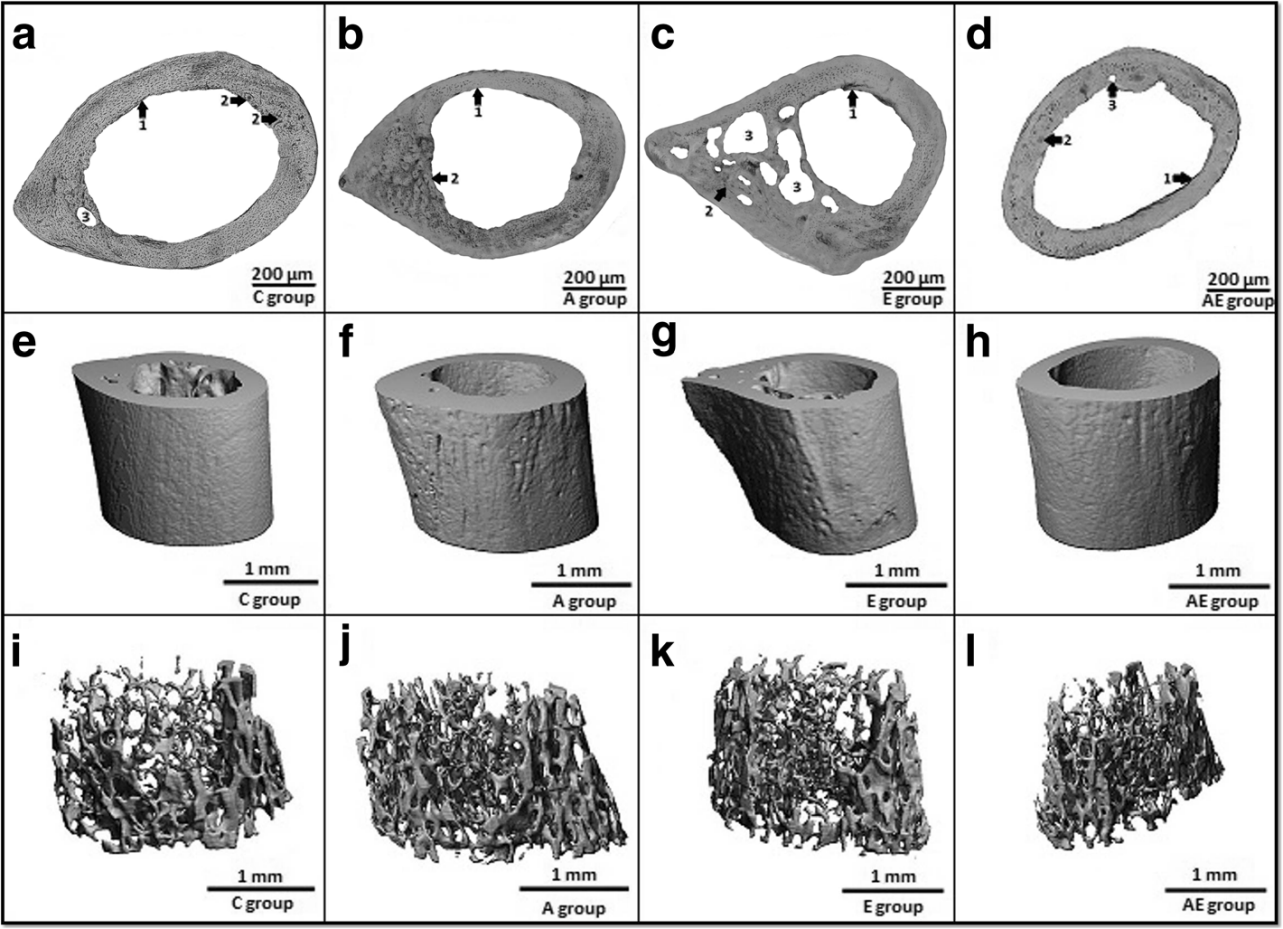
Representative 2D and 3D images of cortical and trabecular bone tissues in mice **a** microscopic structure of cortical bone in control mice. **b** microscopic structure of cortical bone in mice receiving 40 mg/kg bw acrylamide. **c** microscopic structure of cortical bone in mice receiving 15% ethanol. **d** microscopic structure of cortical bone in mice receiving both toxins.1 non-vascular bone tissue. 2 isolated intact secondary osteons. 3 resorption lacunae. **e** representative reconstructed 3D image of the cortical bone in control mice. **f** representative reconstructed 3D image of the cortical bone in mice receiving 40 mg/kg bw acrylamide. **g** representative reconstructed 3D image of the cortical bone in mice receiving 15% ethanol. **h** representative reconstructed 3D image of the cortical bone in mice receiving both toxins. **i** representative reconstructed 3D image of the trabecular bone in control mice. **j** representative reconstructed 3D image of the trabecular bone in mice receiving 40 mg/kg bw acrylamide. **k** representative reconstructed 3D image of the trabecular bone in mice receiving 15% ethanol. **l** representative reconstructed 3D image of the trabecular bone in mice receiving both toxins

Endosteal and periosteal surfaces of femurs in mice from C group consisted of non-vascular bone tissue. In lateral parts near endost, irregular Haversian bone tissue has also been identified. In middle parts of the cortical bone, several secondary osteons were observed. Non-vascular bone tissue was found only in medial parts (Fig. 1a).

Acrylamide-fed mice displayed increased endocortical remodeling. We identified more intact secondary osteons (about 53%) mainly in cranial parts near endosteal surfaces in the A group (Fig. 1b).

A higher number of intact secondary osteons (about 46%) was also observed in cranial parts near endost in mice exposed to ethanol. In addition, four times more resorption lacunae were found in the E group (Fig. 1c) which would be consistent with an increased cortical porosity due to ethanol administration.

The highest density of intact secondary osteons (about 93%) was observed in cranial parts of the cortical bone in mice from the AE group. We identified some resorption lacunae in cranial parts near endosteal surfaces, however their number was expressively lower as compared to the E group.

### Quantitative 2D analysis of cortical bone tissue

Altogether, 692 primary osteons’ vascular canals, 89 Haversian canals and 89 secondary osteons were measured (Table 1). All measured parameters of the primary osteon’s vascular canals and Haversian canals were significantly decreased in acrylamidated mice when compared to the control ones. On the contrary, mice exposed to ethanol had significantly higher values of primary osteon’s vascular canals. However, the sizes of Haversian canals and secondary osteons were significantly decreased in the E group in comparison with the C group. In mice exposed to both toxins (AE group), the values of primary osteon’s vascular canals and Haversian canals were significantly lower as in the C group. Similarly to the A group, the sizes of secondary osteons were not affected by simultaneous exposure to both toxins.

**Table 1:** Quantitative 2D analysis of cortical bone tissue

Measured structures	Group	n	Area (μm^2^)	Perimeter (μm)	Mean. diameter (μm)
Primary osteons’ vascular canals	C (1)	202	31.237 ± 4.453	19.841 ± 1.387	3.152 ± 0.275
	A (2)	160	24.223 ± 3.802	17.484 ± 1.377	2.761 ± 0.271
	E (3)	190	37.823 ± 5.445	21.814 ± 1.569	3.475 ± 0.312
	AE (4)	140	27.038 ± 5.113	18.426 ± 1.751	2.915 ± 0.325
	Games-Howell’s test		1:2*;1:3*;1:4*; 2:3*;3:4*;2:4*	1:2*;1:3*;1:4*; 2:3*;3:4*;2:4*	1:2*;1:3*;1:4*; 2:3*;3:4*;2:4*
Haversian canals	C (1)	15	25.393 ± 4.083	18.027 ± 1.412	2.842 ± 0.301
	A (2)	23	21.311 ± 4.681	16.331 ± 1.821	2.575 ± 0.332
	E (3)	22	22.009 ± 3.639	16.681 ± 1.382	2.625 ± 0.275
	AE (4)	29	19.061 ± 4.535	15.457 ± 1.813	2.431 ± 0.331
	Games-Howell’s test		1:2*; 1:3*; 1:4*	1:2*; 1:3*; 1:4*	1:2*; 1:3*; 1:4*
Secondary osteons	C (1)	15	296.011 ± 43.926	61.227 ± 4.357	9.725 ± 0.895
	A (2)	23	267.056 ± 51.369	58.796 ± 5.425	9.275 ± 0.971
	E (3)	22	248.959 ± 55.878	56.041 ± 6.321	8.885 ± 1.112
	AE (4)	29	250.676 ± 47.697	56.535 ± 5.397	8.951 ± 1.045
	Tukey’s test		1:3*	1:3*	1:2*;1:3*;2:3*;1:4*

*n* number of measurements, *C* control mice, *A* mice receiving acrylamide, *E* mice receiving ethanol, *AE* mice receiving both toxins; *P* < 0.05 (*)

### Quantitative 3D analysis of cortical bone tissue

**Fig. 2:**
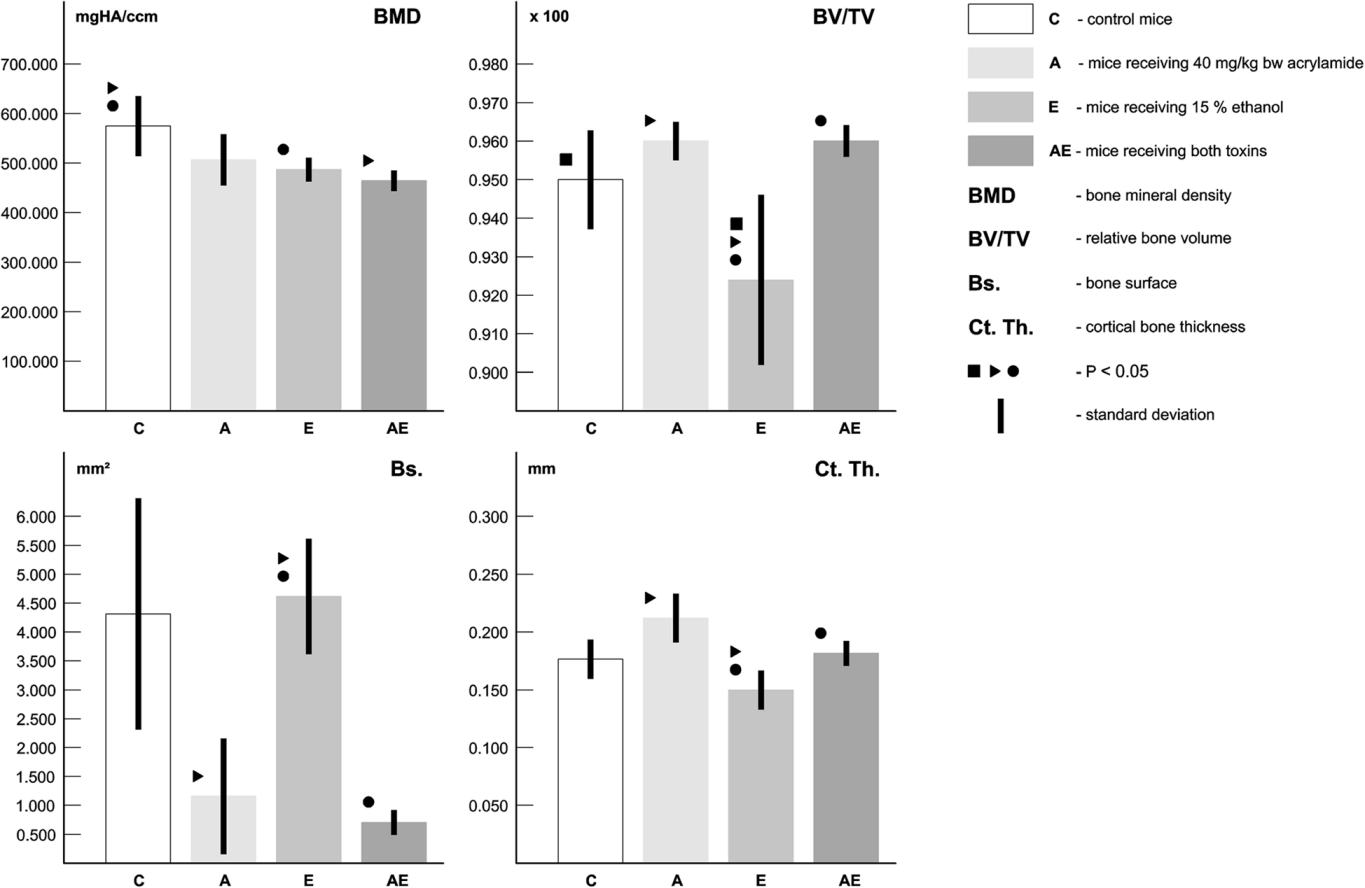
Quantitative 3D analysis of cortical bone tissue

Quantitative 3D analysis of the cortical bone discovered significantly decreased values for relative bone volume and BMD in mice administered ethanol. Significantly lower value for BMD has also been identified in mice from the AE group as compared to the C group. On the other hand, all measured 3D parameters of the cortical bone did not differ significantly between mice from A and C groups (Table 2, Fig. 2). Representative reconstructed 3D images of the cortical bone are illustrated in Fig. 1e, f, g and h.

**Table 2:** Quantitative 3D analysis of cortical bone tissue

Group	n	BMD (mg HA/ccm)	BV/TV (%)	Bs. (mm^2^)	Ct.Th. (mm)
C (1)	5	579.541 ± 61.011	0.951 ± 0.012	4.291 ± 2.092	0.181 ± 0.014
A (2)	5	504.534 ± 54.581	0.961 ± 0.012	1.141 ± 1.114	0.211 ± 0.022
E (3)	5	490.332 ± 23.231	0.924 ± 0.024	4.561 ± 0.962	0.151 ± 0.011
AE (4)	5	470.051 ± 14.734	0.961 ± 0.012	0.704 ± 0.232	0.194 ± 0.012
ANOVA - test		1:3*;1:4*	1:3*;2:3*;3:4*	2:3*;3:4*	2:3*;3:4*

*n* number of measurements, *C* control mice, *A* mice receiving acrylamide, *E* mice receiving ethanol, *AE* mice receiving both toxins, *BMD* bone mineral density, *BV/TV* relative bone volume, *Bs.* bone surface, *Ct. Th.* cortical bone thickness; *P* < 0.05 (*)

### Quantitative 3D analysis of trabecular bone tissue

**Fig. 3:**
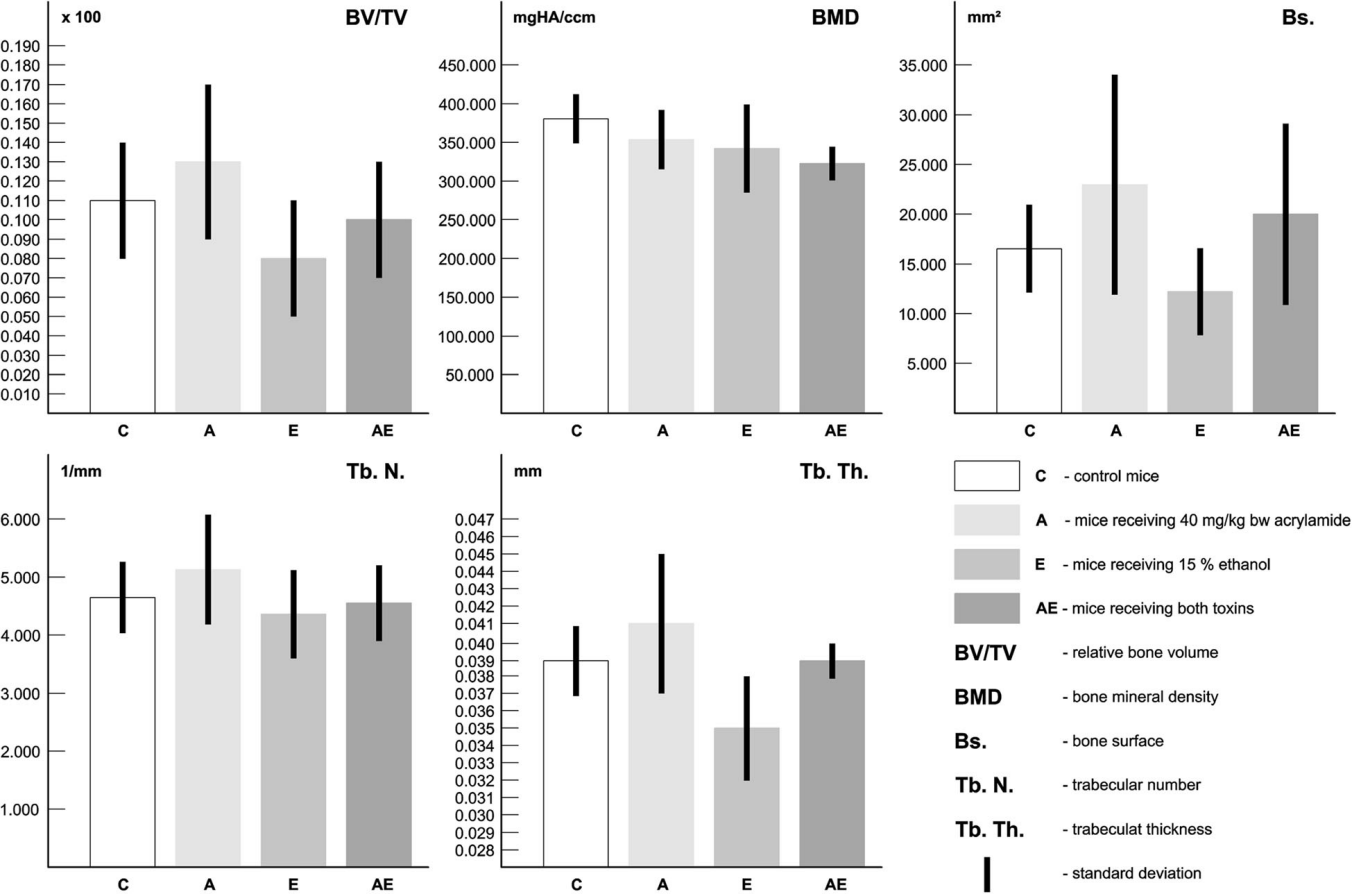
Quantitative 3D analysis of trabecular bone tissue

Trabecular bone microstructure did not differ significantly among mice from all groups. The results are documented in Table 3, Fig. 3. Representative reconstructed 3D images of the trabecular bone are illustrated in Fig. 1i, j, k, l.

**Table 3:** Quantitative 3D analysis of trabecular bone tissue

Group	n	BV/TV (%)	BMD (mgHA/ccm)	Tb.N. (1/mm)	Tb.Th. (mm)	Bs. (mm^2^)
C (1)	5	0.113 ± 0.032	381.843 ± 33.092	4.712 ± 0.591	0.042 ± 0.011	16.473 ± 4.351
A (2)	5	0.132 ± 0.043	357.931 ± 38.092	5.083 ± 0.952	0.0432 ± 0.011	23.43 ± 10.811
E (3)	5	0.083 ± 0.032	347.773 ± 55.382	4.412 ± 0.741	0.041 ± 0.012	12.553 ± 4.291
AE (4)	5	0.103 ± 0.032	319.472 ± 23.461	4.573 ± 0.642	0.043 ± 0.012	20.042 ± 9.091
ANOVA - test		NS	NS	NS	NS	NS

*n* number of measurements, *C* control mice, *A* mice receiving acrylamide, *E* mice receiving ethanol, *AE* mice receiving both toxins, *BV/TV* relative bone volume, *BMD* bone mineral density, *Tb. N.* trabecular number, *Tb. Th.* trabecular thickness, *Bs.* bone surface, *NS* non-significant differences

### Biochemical analysis

**Fig. 4:**
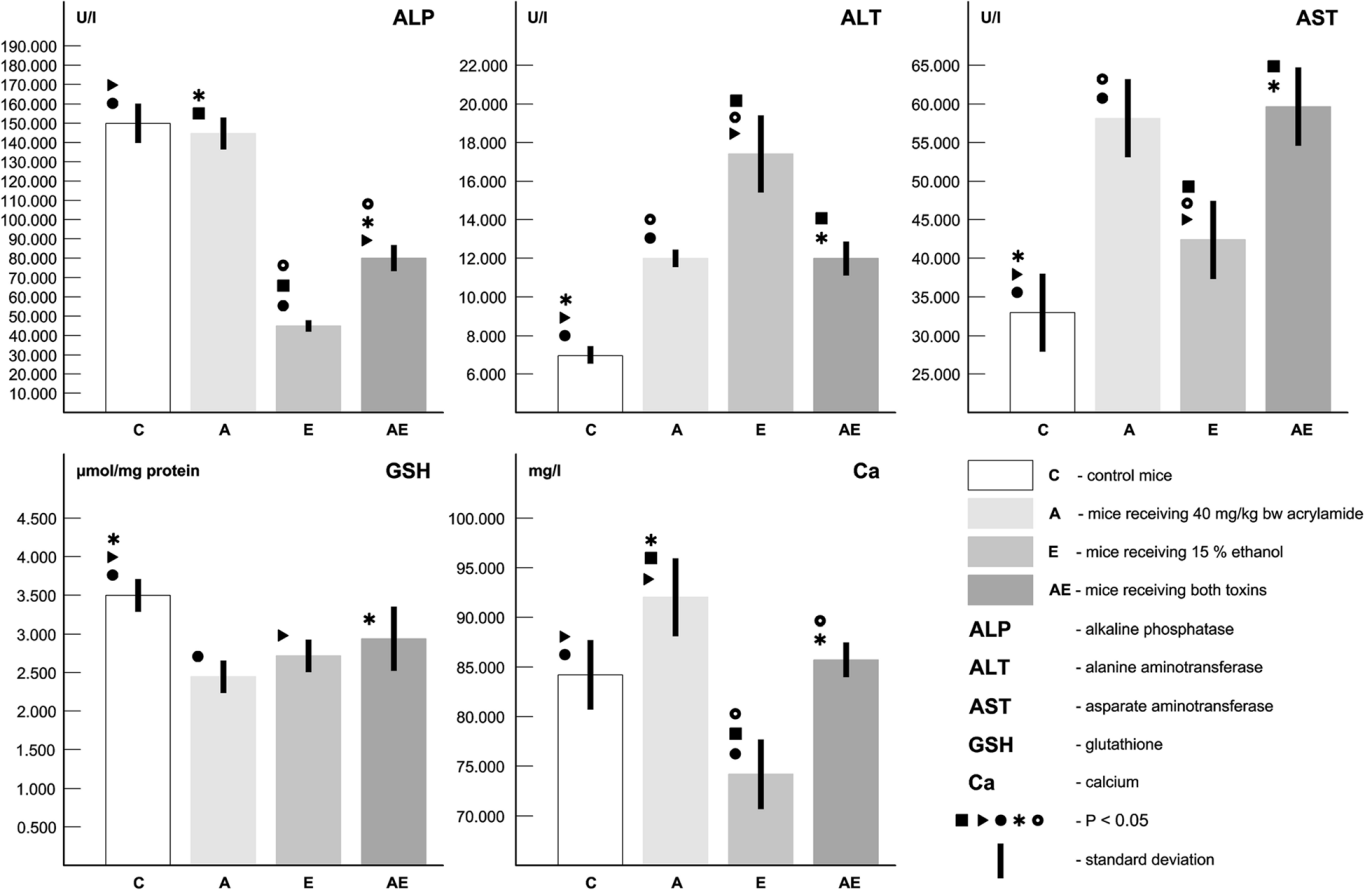
Biochemical analyses

Increased levels of ALT, AST, Ca and decreased value of GSH were documented in acrylamidated mice (Table 4, Fig. 4). In mice exposed to ethanol, similar results were obtained. In addition, the levels of ALP and Ca were significantly decreased in the E group. In the AE group, lower values for ALP, GSH and higher values for ALT, AST were observed (Table 4, Fig. 4).

**Table 4:** Biochemical analyses

Group	n	ALP (U/l)	ALT (U/l)	AST (U/l)	GSH (μmol/mg protein)	Ca (mg/l)
C (1)	5	152.441 ± 9.902	7.123 ± 0.561	32.811 ± 5.483	3.512 ± 0.214	83.941 ± 3.501
A (2)	5	144.162 ± 8.312	12.101 ± 0.521	58.371 ± 5.972	2.441 ± 0.243	92.101 ± 4.342
E (3)	5	44.292 ± 2.601	17.511 ± 1.742	42.321 ± 5.721	2.672 ± 0.212	73.661 ± 2.863
AE (4)	5	79.612 ± 6.821	11.952 ± 1.331	59.132 ± 4.721	2.921 ± 0.362	84.911 ± 3.743
ANOVA - test		1:3*;1:4*; 2:3*;2:4*; 3:4*	1:2*;1:3*; 1:4*;2:3*; 3:4*	1:2*;1:3*; 1:4*;2:3*; 3:4*	1:2*;1:3*; 1:4*	1:2*;1:3*; 2:3*;2:4*; 3:4*

*n* number of measurements, *C* control mice, *A* mice receiving acrylamide, *E* mice receiving ethanol, *AE* mice receiving both toxins, *ALP* alkaline phosphatase, *ALT* alanine aminotransferase, *AST* asparate aminotransferase, *GSH* glutathione, *Ca* calcium; *P* < 0.05 (*)

## Discussion

The results of qualitative 2D analysis of the cortical bone in mice from the C group are consistent with those of other researches [26, 27].

Mice exposed single and simultaneously to both toxins displayed differences related to these characteristics. Increased endocortical remodeling presented in acrylamide-fed mice could be associated with a higher level of serum calcium (Table 1). According to Kuchay et al. [28], hypercalcemia might be due to chronic liver disease per sé. An increased values of serum ALT, AST and ALP in rats exposed to AA (5 mg AA/kg bw, for 45 days) had been observed in the study of Alwan et al. [29]. On the other hand, a decrease of GSH level in A group indicates a presence of oxidative stress. It is generally known that AA increases oxidative stress which is associated with an increased level of reactive oxygen species (ROS) which enhnace bone resorption and osteoclastogenesis [30]. However, in our study, significantly increased cortical porosity was observed only in mice receiving ethanol which corresponds with a lower levels of ALP, GSH and Ca in the E group. Generally, liver dysfunction is associated with vitamin D deficiency [31]. Ethanolʼs toxic skeletal effects have been suggested to involve impaired vitamin D/calcium homeostasis [32]. Simultaneous administration to both toxins led to the highest density of intact secondary osteons and occurence of some resorption lacunae which would be consistent with a decreased levels of ALP and GSH. In contrast to the A group, significantly lower values for ALP and Ca were documented in the AE group. Similarly, decreased level of ALT and increased values for ALP, AST were found in the AE group as compared to the E group. These facts could indicate possible antagonistic effect of both toxins on cortical bone structure.

Our results also revealed a vasoconstriction of primary osteons’ vascular canals in acrylamidated mice and, on the other hand, their vasodilation in mice receiving ethanol. Blood vessels present in vascular canals provide nutrition for the bone [33] and can adapt its structure (vascular remodeling) in response to continuous functional changes [34]. In general, AA decreases the high density lipoprotein (HDL) [28]. Low HDL is associated with narrowing or blockage of the arteries and vessels [35]. On the contrary, ethanol has a significant effect on cardiovascular system including peripheral vasodilation [36]. An antagonistic effect of both toxins on the size of primary osteons’ vascular canals has also been identified in our study. However, similar results were not obtained for Haversian canals’ parameters. Simultaneous application of both toxins caused their vasoconstriction. It is generally known that the structure of primary and secondary osteons is different. Haversian canals found in secondary osteons are surrounded by a cement line [24] which does not outlines vascular canals found in primary osteons. Therefore, the cement line could be the main reason for different results in histomorphometry of both canals. According to our results, the size of secondary osteons was affected only by ethanol exposure. This fact could be associated with a decreased bone mineralization which was also demonstrated in our study and also in ethanol-fed rats (3, 6, 13, and 35% ethanol, for 4 month) [37], (36% ethanol, for 42 days) and alcohol-fed mice (10–36% ethanol, for 78 days) [32, 38] (significantly lower values for BMD were documented). Decreased values of relative bone volume were also obtained by other authors [32, 38]. Generally, decreased bone formation rate followed by a low bone mass and decreased BMD are often identified in alcoholics [39]. Significantly lower BMD has also been observed in mice simultaneously exposed to both toxins.

Interestingly, trabecular bone microstructure did not differ significantly among all groups. Although trabecular bone is more actively remodeled than cortical one, it has much larger surface to volume ratio [40] and therefore the duration of one remodeling cycle in cortical bone is shorter than in trabecular bone. Eriksen [41] assumed that the mechanisms of bone remodeling were different in trabecular versus cortical bone, i.e. the cells needed for bone remodeling in trabecular bone travel directly from red marrow to bone surfaces, while cells reached cortical remodeling sites via the vasculature.

In general, acrylamide and ethanol have been employed as experimental probes to investigate biochemical and morphological changes in the liver of rats and mice [42, 43]. The liver disease caused by these toxins was also presented in other studies and was associated with an increased levels of ALT, AST in rats exposed to AA (10 mg/kg bw for 21 days) [42] and mice exposed to ethanol (5–6% ethanol, for 10 days to 12 weeks) [44]. Since the liver produces various molecules that can act as growth factors or hormones, it has been further postulated that a damage of liver function would results in osteoporosis, through affecting the production of bone-active liver molecule [45]. Similarly, in our study, mice single and simultaneously exposed to both toxins had an increased levels of AST, ALT, which reflect cellular damage. Proteins and enzymes are released into the circulatory fluid (e.g., serum) when cell membrane integrity is damaged as a result of toxemia [46]. Also a presence of reduced GSH, one of the essential compounds for maintenance of cell integrity because of its reducing properties and participation in the cell metabolism [47], could lead to the damage of hepatocytes in all experimental groups. In the E and AE groups, the hepatotoxic effect was increased which would be consistent with a decreased levels of ALP. Broulik et al. [48] also reported a lower ALP in ethanol-fed rats (7.6 g 95% ethanol/kg bw for 12 weeks). Negative changes of hepatocytes could also be associated with a lower level of serum calcium in the E group. On the other hand, the increase of serum calcium value in the A group may be consistent with abnormalities in gene expression. It is known that AA can form adduct with the hepatocytes DNA. Generally, liver disease observed in our study can also be implicated with other factors inducing bone damage through the circulation (i.e. RANKL/OPG, vitamin D metabolism, IGF-I).

There are several limitations to our study. First, a relatively high doses of acrylamide and ethanol were used. It would be necessary to find out if lower doses had the same effects. Second, we investigated only subacute effects of these toxins on bone microstructure (after one remodeling cycle). Therefore, to generalize the results, the treatment period should take a longer time including more remodeling cycles. Third, only males were used. To avoid the complexity of the subject it is necessary to study these limitations in separate studies.

## Conclusions

In summary, only changes in cortical bone structure of mice following one remodeling cycle were observed due to single administration of acrylamide and ethanol. However, more pronounced negative effects of ethanol were partly reduced by simultaneous application of acrylamide. Therefore, possible antagonistic impact of these toxins on the structure of the cortical bone was identified in our study.

## Data Availability

The datasets supporting the conclusions of this article are included within the article. The raw data can be requested from the corresponding author.

## Abbreviations

ALP: Alkaline phosphatase
ALT: Alanine aminotransferase
AST: Aspartate aminotransferase
BMD: Bone mineral density
Bs.: Bone surface without marrow cavity
BV/TV: Relative bone volume
bw: body weight
Ca: Calcium
Ct.Th.: Cortical bone thickness
g: gram
GSH: Glutathione
kg: kilogram
mm: millimetre
N: Number of measurements
NS: Non-significant differences
ROS: Reactive oxygen species
SD: Standard deviation
Tb.N.: Trabecular number
Tb.Th.: Trabecular thickness
μCT: microcomputed tomography
μm: micrometre
